# Genetic Dissection and Simultaneous Improvement of Drought and Low Nitrogen Tolerances by Designed QTL Pyramiding in Rice

**DOI:** 10.3389/fpls.2018.00306

**Published:** 2018-03-09

**Authors:** Bo Feng, Kai Chen, Yanru Cui, Zhichao Wu, Tianqing Zheng, Yajun Zhu, Jauhar Ali, Bingbing Wang, Jianlong Xu, Wenzhong Zhang, Zhikang Li

**Affiliations:** ^1^Rice Research Institute, Shenyang Agricultural University, Key Laboratory of Northern Japonica Rice Genetics and Breeding, Ministry of Education, Shenyang, China; ^2^Institute of Crop Sciences, National Key Facility for Crop Gene Resources and Genetic Improvement, Chinese Academy of Agricultural Sciences, Beijing, China; ^3^Shenzhen Institute of Breeding and Innovation, Chinese Academy of Agricultural Sciences, Shenzhen, China; ^4^International Rice Research Institute, Los Baños, Philippines; ^5^Huazhi Rice Bio-Tech Co., Ltd., Changsha, China

**Keywords:** drought tolerance, low-nitrogen tolerance, green super rice, quantitative trait locus/loci (QTL), pyramiding

## Abstract

Drought and low nitrogen are the most common abiotic stresses limiting rice productivity in the rainfed rice areas of Asia and Africa. Development and adoption of green super rice (GSR) varieties with greatly improved drought tolerance (DT) and low nitrogen tolerance (LNT) are the most efficient way to resolve this problem. In this study, using three sets of trait-specific introgression lines (ILs) in a *Xian (indica)* variety Huanghuazhan (HHZ) background, we identified nine DT-QTL and seven LNT-QTL by a segregation distortion approach and a genome-wide association study, respectively. Based on performances of DT and LNT and genotypes at the detected QTL, two ILs M79 and M387 with DT and LNT were selected for cross-making to validate the identified QTL and to develop DT and LNT rice lines by pyramiding two DT-QTL (*qDT3.9* and *qDT6.3)* and two LNT-QTL *(qGY1* and *qSF8*). Using four pairs of kompetitive allele specific PCR (KASP) SNP markers, we selected 66 F_2_ individuals with different combinations of the target DT- and LNT-QTL favorable alleles and they showed expected improvement in DT and/or LNT, which were further validated by the significant improvement in DT and/or LNT of their F_3_ progeny testing. Based on evaluation of pyramiding lines in F_3_ lines under drought, low nitrogen (LN) and normal conditions, four promising pyramiding lines having different QTL favorable alleles were selected, which showed significantly improved tolerances to drought and/or LN than HHZ and their IL parents. Our results demonstrated that trait-specific ILs could effectively connect QTL mapping and QTL pyramiding breeding, and designed QTL pyramiding (DQP) using ILs could be more effective in molecular rice breeding for complex quantitative traits.

## Introduction

Rice (*Oryza sativa* L.) is one of the most important crops in the world. It is grown in diverse ecologies worldwide and thus often encounters many abiotic stresses. Among them, drought is one of the most damaging stresses for rice production. Rice is very sensitive to drought, and water shortage in any period of rice growth can cause certain degree of yield reduction. In Asia alone, ∼23 million hectares of rice are rainfed and thus subject to various degrees of drought stress (Wassmann et al., 2009). Breeding high yielding varieties with good levels of tolerance to drought is the best option to enhance rice productivity in drought-prone areas (Li and Xu, 2007).

In the past three decades, pursuing high yield potential has been the number one priority in rice breeding of China and almost all recently released rice cultivars have high yield potentials up to 12 t ha^-1^ or higher under high input (water and fertilizers) conditions in the multi-locational yield trials. Pursuing high yields under high inputs (overuses of fertilizers under well-irrigated conditions) has resulted in dramatically increased incidences of pests and overuses of pesticides. However, the average realized yield of these super rice (hybrid and inbred) cultivars in farmers’ fields nationwide is ∼6.5 t ha^-1^. The primary reason for this huge yield gap is primarily due to the fact that most ‘super’ rice cultivars developed under the high input conditions do not perform well in more than 70% of the moderate- and low-yielding fields, much of which suffer more frequently various degrees of drought and ‘inadequate’ fertilizers. Therefore, there have been efforts to develop green super rice (GSR) varieties that have high and stable yields under less input and various environmental stresses (Ali et al., 2013; Iii et al., 2014).

In developing GSR varieties, plant breeders have to deal with many green traits such as tolerances to different abiotic stresses and nutrient use efficiency, all of which are complex quantitative traits controlled by multiple genes and affected by genetic backgrounds (GBs) and environmental conditions. This makes it difficult to improve these complex traits using conventional breeding approaches. Meanwhile, considerable progresses have been made in genetic and molecular dissection of these complex traits of rice from worldwide efforts in rice functional genomic research in a hope that complex traits could be more efficiently improved through marker-assisted selection (MAS) (Collard and Mackill, 2008; Xu et al., 2012). Unfortunately, in the past decades, DNA markers used in mapping populations were usually in most cases low density SSR markers, resulting in that most identified QTL covered very large chromosomal intervals containing hundreds of genes. Due to low resolution of QTL mapping and existence of gene epistasis (i.e., GB) and QTL by environment interactions, previously identified QTL have been limited applications in improvement of rice DT and LNT. Recently, high-throughput SNP genotyping based on re-sequencing and gene chips promises to greatly accelerate QTL mapping and pyramiding on the whole genome (Thomson, 2014).

QTL mapping strategy has been extensively applied to dissecting the genetic architecture of complex traits (including DT and LNT) in rice for more than two decades. Numerous studies have been carried out to identify QTL affecting rice DT and LNT and related traits at the reproductive stage (Lanceras et al., 2004; Trijatmiko et al., 2014; Sellamuthu et al., 2015). Using grain yield under drought as an indicator of DT, researchers identified some main-effect DT-QTL in rice. These included *qDT2.1* and *qDT3.1* detected using bulk segregant analysis (Venuprasad et al., 2009), *qtl12.1* detected under severe drought stress (Bernier et al., 2007), and *qDT1.1* detected under the reproductive-stage drought stress (Prashant et al., 2011). LNT is defined as plants’ ability to maintain normal growth and good yield when the soil N content is low. Thus, LNT could have something to do with nitrogen use efficiency (NUE) to some extent. So far, many QTL have been identified for LNT related traits employing different mapping populations (Lian et al., 2005; Shan et al., 2005; Tong et al., 2006; Wang et al., 2009; Feng et al., 2010; Wei et al., 2012; Zhao et al., 2014). Zhang et al. (2015) cloned a gene on chromosome 12, *TOND1*, which was reportedly associated with LNT and NUE by regulating the expression or activity of its interacting proteins, and so as to change rice response to nitrogen under low nitrogen condition.

Huanghuazhan (HHZ) is a widely adaptable *Xian* (*indica*) variety from South China with high grain yield and superior grain quality. However, HHZ is sensitive to drought and high temperature stresses at the reproductive stage. In the past decade, we used HHZ as the recipient and initiated an introgression-breeding program for improving its tolerances to multiple abiotic stresses and developed 496 BC_1_ HHZ introgression lines (ILs) with significantly higher yield under drought, salt and/or non-stress conditions through three rounds of phenotypic selection (Ali et al., 2017). In this study, we tried to demonstrate the usefulness of the HHZ ILs in identifying QTL associated with DT and LNT, and in developing superior GSR lines with greatly improved DT and LNT by designed QTL pyramiding (DQP).

## Materials and Methods

### Plant Materials

Three populations of BC_1_F_4_ HHZ ILs were developed for identifying QTL associated with DT and LNT. These included 63 ILs from cross HHZ × Teqing, 68 ILs from cross HHZ × CDR22, and 75 ILs from cross HHZ × OM1723 (Ali et al., 2017). Based on their performances for DT/LNT and their genotypes at the detected QTL, two ILs, M79 and M387 were selected from populations of HHZ × OM1723 and HHZ × Teqing, respectively, and crossed and to develop an F_2_ population for pyramiding different DT- and LNT-QTL alleles. The F_2_ and its derived F_3_ lines of the pyramiding cross were used for confirmation and analysis of the pyramiding effects of different alleles at target QTL from two donors.

### Phenotyping ILs for DT and LNT

Phenotypic evaluations of 206 lines from the above three IL populations were performed under low nitrogen (LN) and drought stresses, and normal nitrogen (NN) conditions in different seasons during 2013–2014 on the farm of Agricultural Genomics Institute, Chinese Academy of Agricultural Sciences in Shenzhen (22°52′N 113°46′E), Guangdong province, China.

Evaluation of the ILs for LNT was conducted under the low nitrogen (LN) stress and normal conditions in three consecutive seasons (i.e., the early season from March to July, and late season from July to November of 2013, and the early season of 2014). In each season, a randomized complete block design was used with two replications for each IL. The LN field was created by planting rice crops in the field with zero nitrogen application (but normal application of P and K fertilizers) for 10 consecutive seasons in the past 5 years. In the normal (non-stress) field, 140 kg N ha^-1^ was applied (∼70% used as basal and 30% applied in 15 days after transplanting). Phosphorus (40 kg ha^-1^) and potassium (40 kg ha^-1^) fertilizers were also applied as basal under stress and normal conditions. The paddy soil in the LN field before the experiment had pH 6.18, organic matter of 4.32 g kg^-1^, total N of 310 mg kg^-1^, available P of 79.2 mg kg^-1^, and available K of 165 mg kg^-1^. Contrarily, the paddy soil in the normal field before the experiment had pH 5.97, organic matter of 10.9 g kg^-1^, total N of 920 mg kg^-1^, available P of 88.8 mg kg^-1^, and available K of 155 mg kg^-1^.

Evaluation of the ILs and the recurrent parent, HHZ for DT was conducted in the water sheltered facility during the late season of 2013. Plots were arranged in a complete block design with 2 replications for each IL. Normal irrigation was maintained for 1 month after transplanting, then the plots were drained and irrigation was withheld completely till harvest. Thus, all tested lines were subjected to severe drought at the reproductive stage. During the periods of drought stress, water levels of the fields were monitored daily in the root zone by tensiometers (EM50 Decagon Devices Inc., Pullman, WA, United States). The evaluation of the ILs under the normal non-stress (check) condition was conducted in the same way as the LNT experiment.

In each experiment, seeds of the ILs were sown on the seedling bed and 30 30-day-old seedlings (early seasons of 2013 and 2014) and 23-day-old seedlings (late season of 2013) of each IL were transplanted into a 3-row plot (10 plants per row) at a spacing of 20 cm × 25 cm. One plot of HHZ was inserted every 10 plots as checks in the fields of both stress (LN and drought) and non-stress conditions. All field managements followed the standard local farmers’ practices. At the maturity stage, eight plants in the middle row of each plot were measured for six traits, including heading date (HD, days), plant height (PH, cm), panicle number per plant (PN), filled grain number per panicle (FGN), grain yield per plant (GY, g), 1000-grain weight (TGW, g) and spikelet fertility per panicle (SF, %) according to the standard evaluation system (IRRI, 1996). In the experiment for measuring LNT, the nitrogen stress index was calculated for each yield related trait collected from each plot as the ratio of the trait measured under the LN stress to the trait under the non-stress condition.

### Detection of QTL for DT and LNT

A total of 400 K high-quality SNPs were previously developed based on whole genome re-sequencing of the parents of the ILs (HHZ and eight donor parents) (Zhu et al., 2015). From these SNPs, 41,754 high-quality SNPs were selected and used for QTL mapping (Zhu et al., 2015). Two strategies were adopted for detecting QTL associated with DT and LNT in this study. The first one was the approach of selective introgression (Zhang et al., 2014; Wang et al., 2015). Of 63, 68, and 75 ILs from crosses HHZ × Teqing, HHZ × CDR22, and HHZ × OM1723, 18, 18, 21 ILs were selected once or twice for improved yield under severe drought (Ali et al., 2017). So, these DT-ILs were used for detecting DT QTL via segregation distortion (MSD) approach (Cui et al., 2015). The principle underlying this approach was that when a segregating population was subject to strong directional selection for DT, markers linked to genes controlling DT were expected to show distorted segregation compared to the Mendelian ratio. Because breeding populations after selection tended to have small sample sizes, particularly when selection intensity is high, the power of MSD detection can be low from a single population. Thus, we combined all DT-ILs selected from the above three populations together and performed a joint analysis for MSD in multiple populations to increase the statistical power (Cui et al., 2015). Consensus markers, i.e., the same genotypic loci in different populations, were used to test segregation distortion via generalized linear mixed model (GLMM). In the GLMM, the genetic effect of each identified QTL was estimated to identify whether the locus was segregated with the expected Mendelian frequencies of 23/32AA : 2/32AB : 7/32BB in BC_1_F_4_, where A, H, B represent homozygous genotypes of the recurrent parent (HHZ), heterozygous genotype, and homozygous donor genotypes, respectively. The detailed principle of segregation distortion method for QTL mapping was described in Supplementary Data Sheet 1.

Low nitrogen tolerance is a non-target trait that was not selected during the development of the IL populations. Thus, the selected ILs populations could be roughly considered as random segregation populations for mapping QTL affecting LNT, assuming LNT was not correlated with those selected traits (tolerances to drought, salt and submergence). Association analyses were conducted in the combined three selected populations using a random model by treating the founder effects of each locus as random effects following a normal distribution with a locus-specific variance. The population structure was considered by defining an *n* × 4 matrix of founder allele inheritance indicator for locus *k* and the kinship matrix was used to estimate the polygenic effect to reduce spurious association (Zhu et al., 2015; Wei and Xu, 2016). Let *y* be an *n* × 1 vector for the phenotypic values of *n* individuals. *Z_k_* is an *n* × 4 matrix of the founder allelic indicators for locus *k*. *Z*_jk_ is defined as a 1 × 4 vector of allelic indicators for individual *j*. If the individual is a homozygote and both alleles from the first founder, the *Z*_jk_ is defined as *Z*_jk_ = [2 0 0 0]. If the individual is a heterozygote carrying the second and third alleles, the *Z*_jk_ is defined as *Z*_jk_ = [0 1 1 0]. The sum of all four elements in *Z*_jk_ equals to 2. The random model for testing the significance of the *k*th marker is defined as y = Xβ + Z_k_γ_k_ + ξ + 𝜀, where *Z*_k_ is the inheritance indicator for marker *k*, γ_k_ is a 4 × 1 vector for the four founders allelic effects. ξ is the polygenic effect and 𝜀 is the residual error. The Wald test statistic of each marker was calculated and *P*-value was obtained from chi-square distribution with 3 degrees freedom. The threshold to declare a significant association was set at a probability level of 1.0 × 10^-4^ (Matthus et al., 2015; Wissuwa et al., 2015). An LD block harboring significant SNPs was then defined as a putative QTL. The detailed principle of mixed model approach for QTL mapping was described in Supplementary Data Sheet 1.

### Validation of DT- and LNT-QTL Using the Pyramiding F_2_ Population

Before implementing pyramiding of main-effect QTL of DT and LNT, it was necessary to validate the detected QTL using the F_2_ population derived from M79 × M387. Based on the genotypes between the pyramiding parents, it was clearly aware of the polymorphic regions and polymorphic Kompetitive Allele Specific PCR (KASP) markers at target QTL intervals. We checked all polymorphic KASP markers located in the intervals. Among them, two polymorphic KASP markers closest to each target QTL were used to genotype the F_2_ individuals under the two stress (LN and drought) conditions. Herein, the interval genotype delimited by two polymorphic KASP markers flanking the target QTL represented the QTL genotype. All individuals were then divided into two groups based on the interval genotype, i.e., homozygous genotype of OM1723 or Teqing alleles and homozygous genotype of HHZ alleles. At maturity, GY and SF were individually investigated in the two groups under drought and LN conditions. A *t*-test was performed to find the significant differences in the means of GY and SF between the two homozygous groups under drought and LN conditions. Significant differences indicated that the QTL was true.

### Developing DT and LNT Lines by Designed QTL Pyramiding

We developed a pyramiding F_2_ population by crossing two ILs, M79 and M387, selected based on their performances of DT and LNT, genotypes at four target QTL (*qDT3.9*, *qDT6.3*, *qGY1*, and *qSF8*) and recovery of the HHZ genome based on 3,162 KASP SNP markers differentiating the pyramiding parental ILs. The F_2_ population of the pyramiding cross was divided into three subpopulations each having 160 individuals and being planted under the drought, LN, and NN conditions, respectively, and measured for grain yield and components in the same way as the IL population described above. HHZ, M79, M387 and their derived 480 F_2_ individuals were genotyped by 3,162 evenly distributed KASP SNP markers using the LGC SNPline system by Huazhi Rice Bio-Technology Company^1^ (Supplementary Figure S1). Individuals with different QTL and their combinations can be selected using their respective interval genotypes delimited by the two linkage KASP SNP markers at each QTL. Individuals pyramiding different DT and/or LNT QTL were harvested for evaluation of GY and its related traits in the F_3_ lines.

### Evaluation of Pyramiding Lines for DT and LNT

In the late season of 2016, 66 F_3_ lines selected based on the QTL genotypes of the F_2_ plants were progeny tested and measured for yield related traits under drought, LN, and NN conditions on the farm of Agricultural Genomics Institute, Chinese Academy of Agricultural Sciences in Shenzhen. The same experimental design and field management were adopted as the ILs described above except for three replications for each of the pyramiding lines carrying two of the target QTL, the parents and grandparents, and five replications for the pyramiding lines with three of the target QTL. Genotypes of all selected F_3_ lines at the target QTL were also confirmed by their tightly linked flanking KASP SNPs. At maturing stage, GY and its component traits (HD, PH, PN, FGN, TGW, and SF) were measured. Duncan’s multiple range tests were employed to determine significant differences in mean values of the measured traits among different pyramiding lines, the parents and grandparents.

## Results

### Performances of ILs Under Low Nitrogen (LN) and Drought Conditions

Under the LN conditions, HHZ had GY of 10.7, 10.1, and 9.8 g in the 2013ES, 2013LS, and 2014ES, with yield reductions by 52.9, 51.9, and 57.0% than that under the non-stress conditions, respectively. Although the three IL populations had average GY and yield traits similar to the recurrent parent, HHZ, across different seasons under both the LN and normal conditions (Supplementary Table S1), the ILs segregated significantly for almost all measured yield traits. Many lines showed significantly higher GY than HHZ across the three seasons. These included 7–13 ILs from population HHZ × OM1723; 4–8 lines from population HHZ × Teqing, and 6–9 lines from population HHZ × CDR22 across the three seasons. From these ILs, we identified three promising lines, M79 from HHZ × OM1723, M387 from HHZ × Teqing, and M497 from HHZ × CDR22 that showed consistently higher GY under the LN stress and normal conditions across the three seasons.

Among the 63, 68, and 75 ILs, 18, 18, and 21 DT ILs were selected for DT in the first round of screening then progeny tested in the second round under drought stress from HHZ × Teqing, HHZ × CDR22, and HHZ × OM1723, respectively (Ali et al., 2017). These DT ILs were thus used for identifying QTL associated with DT using the method of selective introgression (Zhang et al., 2014). Under drought stress, HHZ had GY of 11.2 g per plant (Supplementary Table S2), a reduction by 13.4 g (54.5%) when compared with the normal conditions, indicating that drought stress was moderately severe. For the 18 ILs from cross HHZ × Teqing, the drought stress caused an average yield reduction by 10.8 g (39.7%), which was associated with 5.1 days (5.9%) delayed heading (HD), 17.0 cm (18.0%) reduced height (PH), 3.1 (22.1%) reduced panicle number per plant (PN), 42.0 (23.5%) filled grain number per panicle (FGN), 2.4 g (10.8%) thousand grain weight (TGW), and 24.7% (28.6%) reduced seed fertility (SF). For the 18 ILs from cross HHZ × CDR22, drought caused an average yield reduction by 8.0 g (33.9%), and associated reductions in HD by 4.4 days (5.2%), PH by 9.7 cm (11.6%), PN by 5.6 (35.7%), FGN by 38.3 (24.8%), TGW by 1.5 g (6.5%), SF by 23.6% (27.5%). Similarly, the average yield reduction by drought in the 21 ILs from cross HHZ × OM1723 was 8.8 g (34.5%), which was associated with delayed HD by 7.3 days (8.6%), reduced PH by 18.8 cm (18.4%), reduced PN by 4.2 (27.8%), reduced FGN by 38.2 (23.1%), reduced TGW by 2.6 g (10.4%), and reduced SF by 22.5% (26.1%). Comparatively, most DT ILs had significantly higher GY than HHZ under both the drought and normal conditions. GY was highly and positively correlated with FGN and PN in the three IL populations under all the drought, LN, and normal conditions, indicating that FGN and PN were two key components determining GY in the three populations. The correlation between the same traits across stress (drought and LN) and normal conditions was very low in all three ILs populations, indicating strong G × E interactions for the measured traits.

### Identification of QTL for DT

Nine QTL for DT were detected and mapped to chromosomes 2, 3, 5, 6, 8, 12 through joint analysis of segregation distortion using DT-selected ILs from the three populations (**Table 1**). Of these QTL, *qDT6.3* and *qDT8.4* were detected in a single population with the favorable allele for improved DT from the donor, Teqing; *qDT12.3* was detected in two populations with the favorable allele for improved DT either from the donor or from the recipient; and the remaining six QTL were identified in all three populations. At two QTL, *qDT3.1* and *qDT3.9*, the favorable alleles for improved DT were all from the donor, and at *qDT2.8*, *qDT3.8*, *qDT5.4*, and *qDT5.5*, the favorable alleles for improved DT were either from the donor or from the recipient.

**Table 1:** QTL results of DT detection via joint analysis of segregation distortion in three selected breeding populations.

QTL	Chr.	Bin interval	Position^a^ (100 k)	Wald value^b^	Donor genetic effect			Source of favorable allele^c^	QTL/Marker previously reported
					Teqing	CDR22	OM1723		
*qDT2.8*	2	915–917	312–313	10.59	-0.93^∗^	1.08^∗^	0.98^∗^	Teqing	
*qDT3.1*	3	995–1013	0.5–15	31.85	-1.60^∗^	-1.02^∗^	-0.83^∗^	Teqing, CDR22, OM1723	
*qDT3.8*	3	1390	311.5	10.20	-1.67^∗^	0.80^∗^	1.60^∗^	Teqing	
*qDT3.9*	3	1447	350.5	22.96	-1.30^∗^	-0.90^∗^	-1.47^∗^	OM1723, Teqing, CDR22	*QSf3, QGyp3* (Wang et al., 2013a)
*qDT5.4*	5	2208	247	16.72	-0.64^∗^	-1.00^∗^	1.30^∗^	CDR22, Teqing	*QFgwp5b*, *QGyp5* (Wang et al., 2014)
*qDT5.5*	5	2228	261	9.26	-0.54^∗^	0.94^∗^	0.90^∗^	Teqing	
*qDT6.3*	6	2339–2347	37.5–42	11.52	-0.98^∗^	-0.06	0.25	Teqing	
*qDT8.4*	8	3255–3257	206.5–207.5	8.75	-0.74^∗^	0.16	-0.19	Teqing	*QGy8* (Xu et al., 2005); *QPn8*, *QTgw8*, *QSf8* (Wang et al., 2012)
*qDT12.3*	12	4351–4354	29–31.5	11.06	0.19	-0.85^∗^	1.17^∗^	CDR22	*QPn12*, *QSf12* (Wang et al., 2012); *QSnp12* (Wang et al., 2014)

*^a^ Position is reference to the Rice Genome Annotation Project Release 7 (RGAP 7 of Nipponbare). ^b^ P = 0.01, Wald value = 11.34; p = 0.05, Wald value = 7.81. ^c^ The genetic effects indicate that the introgression of the donors resulted in the segregation distortion loci. ^∗^ Indicates the significant difference at P < 0.05.*

### Identification of QTL for LNT by Association Analysis

**FIGURE 1:**
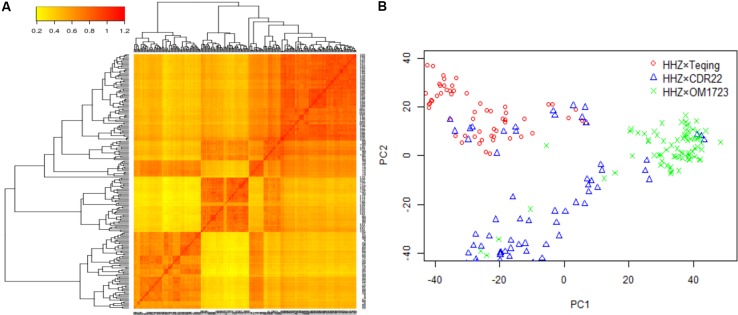
Population structure analysis. **(A)** Heat map of kinship matrix with the tree shown on the top and left. **(B)** Plot of the first two principal components in the selected populations.

The three selected populations were combined to detect the QTL for LNT. Both the heat map of kinship relatedness matrix and the principal component analysis (PCA) showed that there were three distinct subpopulations in this combined population (**Figure 1A**). The PCA results were a little different from the original three populations. A small number of individuals from population HHZ × CDR22 were classified into population HHZ × Teqing, six individuals from population HHZ × OM1723 were classified into population HHZ × CDR22, and six individuals from population HHZ × CDR22 were classified into population HHZ × OM1723 (**Figure 1B**). In our association analysis method, we coded the genotype indicator (Z_jk_) according to the original three populations. The classification result from PCA could be due to the drought selection. However, it is irrelevant to the result of association study.

Using interval mapping, seven QTL for LNT-related traits were identified and mapped to chromosomes 1, 2, 3, and 8 based on a threshold of *P* < 0.0001 (**Table 2**). These included five QTL (*qGY1*, *qPN1*, *qPN2*, *qTGW3*, and *qSF8*) detected under the LN conditions, three of which (*qGY1*, *qTGW3*, and *qSF8*) were also detected under the NN conditions, and two additional QTL (*qRTGW2* and *qRPN8*) detected by the trait ratio under the LN and NN conditions. Of these QTL, two QTL were worth mentioning. The first one was *qGY1* located on the top of chromosome 1. It was detected under both the LN and NN conditions and explained 18.2 and 9.1% of grain yield variances among the ILs. The donor (OM1723) allele at this locus had positive effects of 0.79 and 1.38 g per plant for increased GY under the LN and NN conditions. Interestingly, the Teqing allele at this locus had a positive effect of 0.74 g per plant under NN but a negative effect of -0.46 g per plant for reduced GY under the LN condition. The second one was *qSF8*, which explained 43.3 and 16.4% of the phenotypic variances in seed fertility among the ILs under the LN and NN conditions, respectively. The donor allele from Teqing had a significant effect of 5% for increased seed fertility under the LN condition.

**Table 2:** QTL for yield and yield related traits detected under low-nitrogen (LN) and normal nitrogen (NN) conditions in 3 HHZ IL populations across three seasons during 2013–2014.

Env.	Season^a^	QTL	Chr.	Bin interval	Position (100k)	*P*-value	PVE (%)	QTL effect^b^				Source of the favorable allele^c^	QTL reported previously^d^
								HHZ	OM1723	Teqing	CDR22		
LN	2013LS	*qGY1*	1	16	8.0	4.6E-05	18.2	-0.28	0.79*	-0.46*	-0.05	OM1723	
	2013LS	*qPN1*	1	481–519	403.0–432.5	8.1E-12	8.9	0.09	0.19	-0.02	1.62*	CDR22	*rrw1b, n-p1, n-s1* [1]; *qNUEn1*, *qGYn1* [2]
	2013LS	*qPN2*	2	835–893	258.0–296.5	6.5E-16	15.5	0.02	0.27*	0.24*	1.65*	CDR22	*qSNR2*, *qNPBPE2* [3]
	2013LS	*qTGW3*	3	1294–1308	235.5–246.5	7.3E-05	7.7	-0.79*	-0.29	-0.84*	1.92*	CDR22	*n-p3* [1]
	2013ES	*qSF8*	8	3086–3107	62.5–82.5	1.3E-10	43.3	0.04	-0.13*	0.05*	0.04	Teqing	*qGNA8a* [3]
NN	2013ES	*qGY1*	1	16	8.0	7.7E-05	9.1	0.34	1.38*	0.74*	0.30	OM1723	
	2013ES	*qTGW3*	3	1294–1308	235.5–246.5	4.3E-06	7.6	-0.86*	-0.49	-1.08*	2.44*	CDR22	*n-p3* [1]
	2013ES	*qSF8*	8	3086–3107	62.5–82.5	6.5E-05	16.4	0.01	-0.03*	0.01	0.01	Teqing	*qGNA8a* [3]
LN/	2013ES	*qRGY1*	1	16	8.0	8.4E-05	7.1	-0.01*	0.01*	0.002	-0.004	OM1723	
NN	2013LS	*qRTGW2*	2	852–855	269.0–272.0	8.0E-05	8.4	-0.012*	-0.007	0.013*	0.006	Teqing	*qGNR2b* [3]
	2013ES	*qRSF8*	8	3086–3107	62.5–82.5	1.2E-18	13.1	0.03	–0.10*	0.04	0.03	Teqing	*qGNA8a* [3]
	2013LS	*qRPN8*	8	3009–3012	14.0–16.5	1.5E-06	7.0	-0.02	0.05*	-0.02	-0.01	OM1723	

*^a^ 2013ES: 2013 early season, 2013LS: 2013 later season. ^b^ QTL effect is the trait value associated with the donor allele, and ^∗^indicates the statistical significance at P < 0.05. ^c^ The donor carries the allele for positive trait value. ^d^ [1] = (Lian et al., 2005), [2] = (Wei et al., 2011), [3] = (Li et al., 2010).*

### Validation of DT- and LNT-QTL and Developing Superior DT and Low Nitrogen Tolerant Lines by Designed QTL Pyramiding (DQP)

Based on the above QTL mapping results, four large-effect QTL, *qDT3.9*, *qDT6.3*, *qGY1*, and *qSF8*, were selected as the target QTL. Based on their QTL genotypes at the four target QTL and performances under the drought, LN and NN conditions, two ILs, M79 and M387, were selected for QTL validation and for developing superior lines in the DQP experiment (**Table 3**). M79 and M387 had GY of 16.8 and 15.6 g under drought and ratios of GY 0.64 and 0.61 of the drought to normal conditions, significantly higher than those of HHZ (10.3 and 0.39 g), indicating that M79 and M387 were DT. Also, M79 and M387 had significantly higher average GY of 17.5 and 17.1 g under LN and ratios of GY 0.67 and 0.66 of LN stress to control conditions than those of the HHZ (12.7 and 0.48 g), indicating that M79 and M387 were also LNT. Additionally, M79 possessed 81.4% of the HHZ genome with the OM1723 favorable alleles at *qDT3.9* for DT and *qGY1* for LNT, while M387 possessed 87.1% of the HHZ genome with the Teqing favorable alleles at *qDT6.3* for DT and *qSF8* for LNT. Thus, M79 and M387 had non-allelic complementary favorable alleles at the target DT- and LNT-QTL (**Table 3**).

**Table 3:** Polymorphic KASP SNP genotypes of the parents (M79 and M387) and grandparents (HHZ, OM1723, and Teqing) of the pyramiding cross.

QTL	Chr.	QTL position (100 kb)	KASP SNP marker	Marker position (100 kb)	Trait	Genotype of the parents and grandparents					Favorable allele
						HHZ	OM1723	Teqing	M79	M387	
*qDT3.9*	3	350.5	HZ_Os03273	354.3	DT	G	A	G	A	G	A
			HZ_Os03275	355.1		G	A	G	A	G	A
*qDT6.3*	6	37.5–42.0	HZ_Os06037	37.9	DT	G	G	A	G	A	A
			HZ_Os06038	39.1		A	A	G	A	G	G
*qGY1*	1	8.0	HZ_Os01009	6.6	LNT	A	G	A	G	A	G
			HZ_Os01012	9.5		A	G	A	G	A	G
*qSF8*	8	62.5–82.5	HZ_Os08054	61.1	LNT	G	G	T	G	T	T
			HZ_Os08058	67.5		G	G	T	G	T	T

**Table 4** shows the genotypic frequencies of 480 F_2_ plants from the pyramiding cross M79 × M387 at the four target QTL and the mean trait values of GY and SF of the homozygous F_2_ genotypes (based on flanking KASP markers) at the target QTL under the drought and LN conditions. Under the drought stress, the 34 and 35 homozygous donor (BB) F_2_ plants at *qDT3.9* and *qDT6.3* had average GY of 15.1 and 14.5 g, significantly (52.5 and 40.8%) higher than their corresponding homozygous recipient (AA) F_2_ genotypes. The 37 homozygous donor (BB) F_2_ plants at *qGY1* had an average GY of 15.6 g under the LN condition, significantly (39.3%) higher than their 32 corresponding homozygous recipient (AA) F_2_ genotypes. Similarly, the homozygous donor F_2_ genotypes at *qSF8* had a mean 84.0% of SF, significantly higher than the homozygous HHZ F_2_ plants by 10.1%. Thus, all four target QTL were validated, with *qDT3.9* and *qDT6.3* contributing to DT and *qGY1* and *qSF8* contributing to LNT. Results from **Table 4** also led us to identify 66 F_3_ lines from the pyramiding F_2_ population that were homozygous at the four QTL for progeny testing.

**Table 4:** Validation of DT- and LNT-QTL in the pyramiding F_2_ population derived from M387 × M79 under drought and low nitrogen conditions.

Trait	Environment	*N*	QTL	Trait measured^a^	Favorable allele	KASP marker	AA genotype^b^		BB genotype		AA–BB
							*N*	Mean ±*SD*	*N*	MS ±*SD*	
DT	Drought	160	*qDT3.9*	GY	OM1723	HZ_Os03273–HZ_Os03275	36	9.9 ± 2.9	34	15.1 ± 0.6	-5.2^∗^
DT	Drought	160	*qDT6.3*	GY	Teqing	HZ_Os06037–HZ_Os06038	40	10.3 ± 2.3	35	14.5 ± 3.8	-4.2^∗^
LNT	Low nitrogen	160	*qGY1*	GY	OM1723	HZ_Os01009–HZ_Os01012	32	11.2 ± 3.3	37	15.6 ± 0.9	-4.4^∗^
LNT	Low nitrogen	160	*qSF8*	SF	Teqing	HZ_Os08054–HZ_Os08058	39	76.3 ± 3.1	33	84.0 ± 2.3	-7.7^∗^

*^a^ GY, grain yield (g/plant); SF, seed fertility (%). ^b^ AA = homozygous recipient (HHZ) genotype; BB = homozygous donor (OM1723 or Teqing) genotype. ^∗^ Indicates the significant difference at P < 0.05.*

### Pyramiding Effect of DT- and LNT-QTL on GY and Yield Components

**Table 5** shows the F_3_ progeny testing results. Based on the genotypes at the four target QTL, the F_3_ progeny could be divided into four genotypic groups each containing 2–3 favorable alleles at the target QTL. The 29 F_3_ lines of group 1 had the favorable donor alleles at both DT-QTL (*qDT3.9* and *qDT6.3*) and showed an average GY of 18.8 g, significantly higher than their parents, M79 (16.8 g) and M387 (15.6 g) each having a single DT-QTL and HHZ (10.3 g) under drought stress. The 27 F_3_ lines of group 2 pyramided two different LNT-QTL with the homozygous OM1723 allele at *qGY1* and the Teqing allele at *qSF8*, and had an average GY of 20.8 g, significantly higher than their parents, M79 (17.5 g) and M387 (17.1 g) each having one LNT-QTL and HHZ (12.7 g) under the LN stress. Group 3 consisted of five lines in two subgroups each pyramiding two DT-QTL and one LNT-QTL. Subgroup 3-1 contained three homozygous plants with the donor alleles at both DT-QTL plus a Teqing allele at *qSF8*, while the two subgroup 3-2 lines had the same donor DT alleles but an OM1723 allele at *qGY1*. Phenotypically, both subgroups 3-1 and 3-2 had significantly higher mean GY (19.3 and 19.1 g) than their parental ILs, M79, M387 and grandparent, HHZ under drought stress, but similar GY (16.9 and 17.3 g) to M79 and M387 under LN stress. Group 4 plants pyramided one DT-QTL and two different LNT-QTL, including two plants homozygous for OM1723 allele at *qGY1*, Teqing alleles at *qDT6.3* and *qSF8*, and HHZ allele at *qDT3.9* (subgroup 4-1), and three plants homozygous for OM1723 alleles at *qDT3.9* and *qGY1*, Teqing allele at *qSF8* and HHZ allele at *qDT6.3* (subgroup 4-2). They had significantly higher GY (20.5 and 20.3 g) than those of M79, M387 and HHZ under LN stress and similar GY (16.3 and 16.0 g) with M79 and M387 but significantly higher than that of HHZ under drought stress. This result clearly showed that DT and LNT could be much improved by means of pyramiding of non-allelic DT- and/or LNT-QTL alleles.

**Table 5:** Performance of pyramiding lines under drought, low nitrogen stress, and normal conditions.

Group	QTL genotypes^a^				No. of plants/lines	Under drought condition^b^							Under low nitrogen condition						
	1	2	3	4		HD (days)	PH (cm)	PN	FGN	TGW (g)	SF (%)	GY (g)	HD (days)	PH (cm)	PN	FGN	TGW (g)	SF (%)	GY (g)
HHZ	**-**	**-**	**-**	**-**	10	85.2bc	75.1g	7.3f	65.6d	19.4e	52.2c	10.3d	60.1d	75.2f	4.8c	106.9d	19.0e	79.6a	12.7c
M79	**+**	**-**	**+**	**-**	10	89.0ab	95.7c	9.4bc	97.9b	23.1ab	67.5b	16.8b	71.5ab	99.4a	6.3b	123.6b	21.8a	81.7a	17.5b
M387	**-**	**+**	**-**	**+**	10	80.4bc	87.3ef	7.7f	84.8c	22.7bc	65.8b	15.6c	64.2cd	92.2d	6.0b	128.0ab	21.3abc	81.5a	17.1b
1	**+**	**+**	**-**	**-**	29	82.7bc	98.9b	9.6abc	117.7a	23.7a	70.8a	18.8a	64.5c	91.9d	5.0c	110.6cd	20.5bcd	81.4a	13.3c
2	**-**	**-**	**+**	**+**	27	96.1a	86.2f	8.8de	72.5d	19.5e	53.7c	11.1d	74.4a	87.0e	8.0a	125.8b	20.9abcd	82.2a	20.8a
3-1	+	**+**	**-**	**+**	3	80.7bc	102.8a	9.8ab	120.8a	22.1c	71.0a	19.3a	65.1c	95.3bc	6.0b	127.8ab	20.3cd	81.1a	16.9b
3-2	**+**	**+**	**+**	**-**	2	79.1c	97.7bc	10.0a	120.1a	22.8bc	72.5a	19.1a	63.7cd	96.1b	6.1b	120.9bc	20.1de	82.0a	17.3b
4-1	**-**	**+**	**+**	**+**	2	85.0bc	89.6de	9.1cd	91.4bc	20.9d	66.8b	16.3bc	71.1ab	92.9cd	8.1a	138.1a	20.3cd	80.3a	20.5a
4-2	**+**	**-**	**+**	**+**	3	80.7bc	92.0d	8.5e	87.1c	20.9d	66.0b	16.0bc	67.9bc	95.6bc	8.4a	123.2b	21.5ab	82.8a	20.3a

**Group**	**QTL genotypes**				**No. of plants/lines**	**Under normal condition**													
	**1**	**2**	**3**	**4**		**HD (days)**	**PH (cm)**	**PN**	**FGN**	**TGW (g)**	**SF (%)**	**GY (g)**							

HHZ	**-**	**-**	**-**	**-**	10	75.3b	95.0d	11.5d	164.2bc	23.6d	87.5ab	26.3abc							
M79	**+**	**-**	**+**	**-**	10	83.1a	105.3bc	12.6ab	173.1abc	26.1ab	87.5ab	26.1bc							
M387	**-**	**+**	**-**	**+**	10	75.5b	98.1d	10.1e	160.8c	25.9abc	89.8a	25.7c							
1	**+**	**+**	**-**	**-**	29	79.6ab	111.2a	11.6cd	171.7abc	26.3a	86.5b	26.0bc							
2	**-**	**-**	**+**	**+**	27	81.9a	101.8c	12.1bc	173.3abc	25.1bc	86.6b	26.5abc							
3-1	**+**	**+**	**-**	**+**	3	76.3b	111.9a	12.2b	172.4abc	25.4abc	85.5b	27.4a							
3-2	**+**	**+**	**+**	**-**	2	76.0b	110.6a	12.7ab	176.3ab	24.1d	90.4a	26.6abc							
4-1	**-**	**+**	**+**	**+**	2	79.5ab	109.8ab	13.1a	177.6a	25.0c	88.2ab	26.8abc							
4-2	**+**	**-**	**+**	**+**	3	75.0b	113.9a	12.5ab	174.3ab	25.2bc	85.6b	27.2ab

*^a^ 1, 2, 3, and 4 represent qDT3.9, qDT6.3, qGY1, and qSF8, respectively. “+” Indicate lines with favorable alleles from donor parents,“**-**” indicates lines with alleles from HHZ. ^b^Different letters indicate statistically significant difference in mean trait values at P ≤ 0.05, based on Duncan’s Multiple Range Test.*

As compared the parental ILs, improvement of GY of pyramiding lines mainly resulted from significantly increased FGN and SF for both groups 1 and 3 under drought stress while mainly from significantly increased PN for both groups 2, and 4 under LN stress (**Table 5**). Based on the results, four pyramiding lines, PL6, PL36, PL50, and PL66, were selected as the promising lines that performed significantly better than HHZ under drought stress, LN stress, and normal conditions (**Table 6**).

**Table 6:** The performances of yield related traits of four promising HHZ pyramiding lines under drought, low-nitrogen, and normal conditions developed by designed QTL pyramiding.

Pyramiding lines	Target QTL^a^				Under drought condition^b^							Under low nitrogen condition						
	1	2	3	4	HD (days)	PH (cm)	PN	FGN	TGW (g)	SF (%)	GY (g)	HD (days)	PH (cm)	PN	FGN	TGW (g)	SF (%)	GY (g)
PL6	**+**	**+**	**-**	**-**	84.0	103.5	9.8	116.4^∗^	24.2	70.4^∗^	20.2^∗^	66.0	89.8	5.5	107.4	20.8	83.8	14.0
PL36	**-**	**-**	**+**	**+**	92.0	91.4	8.1	83.4	19.9	57.0	13.0	70.0	89.7	8.1^∗^	139.4	22.7	85.2	21.9^∗^
PL50	**+**	**+**	**-**	**+**	77.5	98.4	10.0	119.0^∗^	22.0	70.6^∗^	20.2^∗^	63.0	94.8	6.7^∗^	142.7	20.3	82.9	16.6^∗^
PL66	**-**	**+**	**+**	**+**	86.0	92.1	9.4	89.0^∗^	20.2	67.8^∗^	16.8^∗^	70.0	94.3	8.0^∗^	128.5	20.2	78.5	20.7^∗^
HHZ(CK)	**-**	**-**	**-**	**-**	85.2	75.1	7.3	65.6	19.4	52.2	10.3	60.1	75.2	4.8	106.9	19.0	79.6	12.7

**Pyramiding lines**	**Target QTL**				**Under normal condition**						
	**1**	**2**	**3**	**4**	**HD (days)**	**PH (cm)**	**PN**	**FGN**	**TGW (g)**	**SF (%)**	**GY (g)**							

PL6	**+**	**+**	**-**	**-**	86.0	109.4	12.8^∗^	177.6	26.5	88.7	28.3^∗^							
PL36	**-**	**-**	**+**	**+**	85.0	100.7	13.9^∗^	190.8^∗^	24.7	88.2	30.2^∗^							
PL50	**+**	**+**	**-**	**+**	77.0	107.6	12.7^∗^	173.8	25.8	86.4	27.7^∗^							
PL66	**-**	**+**	**+**	**+**	79.0	105.8	13.0^∗^	176.9	25.2	90.9	27.9^∗^							
HHZ(CK)	**-**	**-**	**-**	**-**	75.3	95.0	11.5	164.2	23.6	87.5	26.3

*^a^ 1, 2, 3, and 4 represent qDT3.9, qDT6.3, qGY1, and qSF8, respectively. “+” Indicate lines with favorable alleles from donor parents, “**-**” indicates lines with alleles from HHZ. ^b∗^Suggested significant at P ≤ 0.05 calculated by t-test, when compared with the check, HHZ.*

## Discussion

### Application of Trait-Specific ILs in QTL Mapping and Designed QTL Pyramiding (DQP) in Rice

In the rainfed areas of South and Southeast Asian countries where drought and infertile soil are the major factors limiting rice productivity, breeding DT and LNT rice varieties is expected to have significant impact on the food security and poverty alleviation of these countries. Successful crop breeding depends on availability of favorable alleles and germplasm resources. However, for complicated quantitative traits, parental lines of most QTL mapping populations of DT and LNT were different from breeding populations. As a result, QTL mapping information can’t be directly applied to breeding practices due to uncertainty of GB effects on QTL expression and QTL × environment (QE) interaction (Tanksley and Nelson, 1996; Li, 2001). To solve this problem, Li et al. (2005) proposed the selective introgression strategy. This approach included three steps: (1) developing trait-specific ILs as the molecular breeding (MB) material platform by introgressing valuable genes/alleles from diverse donors into elite rice GBs; (2) large scale QTL discovery and allelic mining by tracking genome-wide responses of donor segments to selection; and (3) developing GSR cultivars with multiple green (complex) traits by DQP. This strategy has at least three major advantages over the conventional breeding and MAS. First, each IL set consists of hundreds of ILs in an elite recipient background, which contains large numbers of QTL/alleles for selected target traits from a diverse set of donors (Li and Xu, 2007; Li and Zhang, 2013), providing valuable sources and information of loci and favorable alleles for improving the target traits by QTL pyramiding without worrying

GB and QE effects because the QTL are identified in the target environments and elite varieties to be improved. Second, using selective introgression to identify QTL/alleles affecting target traits is much more powerful and efficient because of the low costs in genotyping and phenotyping small selected populations (Li et al., 2005; Cui et al., 2015; Wang et al., 2015). Third, most importantly, this strategy allows complete integration of QTL mapping and allelic mining with breeding. Thus, once favorable QTL/alleles are identified, they are already in the elite GB to be improved. Nevertheless, mapping QTL affecting target traits such as DT in this study is challenging because of the small population sizes and the reduced trait variances in the selected populations. This can be improved by combining several populations together and performing a joint analysis for segregation distortion locus in multiple populations in the same GBs (Cui et al., 2015). In practice, we noted that when all 206 HHZ ILs derived from the three BC populations were pooled for mapping of non-target trait such as LNT, the introgressed segments were not evenly distributed across genome in the ILs, even though the donor introgression in the ILs covered most rice genome. As a result, the number of QTL detected for each of the non-target traits were fewer than most reported cases using random mapping populations. Nevertheless, as demonstrated in this study, association analysis still can be applied to such breeding populations effectively for identifying QTL affecting non-target traits such as LNT in this study and cold tolerance in our previous studies (Zhang et al., 2014; Zhu et al., 2015). Compared with random mapping populations widely performed before, ILs are not only cost-effective both in phenotyping and genotyping owing to small number of lines, but also easily integrate with MB by DQP for both target and non-target traits based on the performances and distribution of QTL alleles in ILs in elite backgrounds, thus, providing a huge potential application in future plant breeding programs.

### Comparison of DT- and LNT-QTL Across Different Populations

In this study, we identified nine DT loci, five (55.6%) of which were previously reported. Specifically, *qDT3.8* was associated most strongly with a SNP at 31,150 kb on chromosome 3, which is adjacent to an SSR marker, RM571, associated with grain yield under drought stress (Venuprasad et al., 2012). *qDT3.9* with the most significant SNP at 35,050 kb on chromosomes 3 was mapped in the adjacent region harboring *QSf3* for seed fertility and *QGyp3* for grain yield per plant under drought stress (Wang et al., 2013a). *qDT5.4* with the most significant SNP at 24,700 kb on chromosome 5 was mapped in the adjacent region harboring *QFgwp5b* for filled grain weight per panicle and *QGyp5* for grain yield per panicle under drought stress (Wang et al., 2014). *qDT8.4* in the region of 20,650–20,750 kb on chromosome 8, was mapped in the same region of *QGy8* for grain yield (Xu et al., 2005), *QPn8* for panicle number, *QTgw8* for thousand grain weight and *QSf8* for seed fertility (Wang et al., 2012) under drought stresses. *qDT12.3* in the region of 2,900–3,150 kb on chromosome 12, was mapped in the same region of *QPn12* for panicle number, *QSf12* for seed fertility (Wang et al., 2012), and *QSnp12* for spikelet number per panicle under drought stresses (Wang et al., 2014). *qDT6.3* was a new DT-QTL identified and validated in this study. The above mentioned DT-QTL were identified in different mapping populations and diverse environments, providing good candidates for developing DT varieties by MAS.

Of the seven LNT-QTL identified in this study, four QTL (*qGY1*, *qTGW2*, *qSF8*, and *qPN8*) most probably belong to LNT-QTL because they affected the ratio of trait under LN to the trait under NN conditions. Of them, the *qTGW2* was probably related to *qGNR2b*, which was previously identified for grain nitrogen rate (Li et al., 2010) because these two QTL are located in the same region of 26,900–27,200 kb on chromosome 2. The *qSF8*, detected consistently in all three conditions in 2013ES, was probably related to the *qGNA8a*, which was previously identified for grain nitrogen accumulation (Li et al., 2010) because both are located in the same region of 6,250–8,250 kb on chromosome 8. The *qGY1* and *qPN8* were newly identified as LNT-QTL, and the former was simultaneously detected in all three conditions while the latter was solely identified under LN/NN condition. In addition, although *qPN1* and *qPN2* were only identified under LN condition, both are located in regions harboring QTL for LNT- or NUE-related traits. For instance, *qPN1* was most probably related to *rrw1b* for relative root weight, *n-p1* for plant weight, *n-s1* for shoot weight measured at rice seedling stage under LN conditions (Lian et al., 2005), and *qNUEn1* for NUE and *qGYn1* for grain yield under NN condition (Wei et al., 2011) because they are located in the same or adjacent region of 40,300–43,250 kb on chromosome 1. *qPN2* was most probably related to *qSNR2* for straw nitrogen rate, and *qNPBPE2* for the ratio of plant weight to plant nitrogen accumulation measured at maturing stage under LN conditions (Li et al., 2010) because they are all located in the same region of 25,800–29,650 kb on chromosome 2. Whether the *qPN1* and *qPN2* were associated with LNT remains to be confirmed. The *qTGW3*, which was detected in 2013LS under LN condition and 2013ES under NN condition, was associated with *n-p3* for plant weight under low nitrogen stress (Lian et al., 2005). The above results suggest that LNT and NUE in rice share the same genetic basis at least partially. If so, screen under LN condition could be an easy way to develop NUE rice varieties.

### High Throughput SNP Marker Detection Accelerates the Process of QTL Mapping and MAS

SNPs are the most common DNA markers used in plant and animal genetic/genomic research. In rice, several high-throughput SNP genotyping systems are commercially available, including PCR-based systems (such as LGC SNPline and Douglas Array Tape) (Semagn et al., 2014; Thomson, 2014), array-based systems (Affymetrix or Illumina SNP array) (Zhao et al., 2011; Chen et al., 2014), and sequencing-based systems (Thudi et al., 2012; He et al., 2014). Coupling with automation techniques, PCR-based system can be used to survey thousands of samples in a short time-frame. Different from array-based or sequencing-based systems, where SNP markers were either fixed or randomly assayed, PCR-based system is very flexible on selecting SNP markers to be assayed, which make it ideal for MAS and multi-trait pyramiding. In this study, we first screened the parental lines using 3,162 genome-wide KASP SNP markers. Two polymorphic KASP markers flanking each target QTL (**Tables 3**, **4**) were then selected for genotyping the pyramiding population to determine the favorable allele in the progeny. This way, the genotyping cost was greatly reduced and turn over time is shortened. As shown in **Table 5**, the progenies selected by SNP markers to pyramid the two DT-QTL (*qDT3.9* and *qDT6.3*) and two LNT-QTL (*qGY1* and *qSF8*) all resulted in significantly increased GY than their parents. All pyramiding lines in groups 1–4 showed expected increased phenotype, suggesting that our selection based on KASP SNP markers was highly accurate. These results strongly demonstrated the power and effectiveness of high-throughput PCR-based SNP markers in QTL mapping and MAS of rice.

### Implication in Rice Breeding for Multiple Abiotic Stress Tolerances

Registered by 12 provinces in China, HHZ is a mega-variety with good yield potential and excellent grain quality. It also showed wide adaptability in many tropic countries in Asia and Africa (Ali et al., 2017). However, it has lost its popularity because of its susceptibility to many abiotic stresses such as drought, salinity, high temperature, and disease resistance. In addition, HHZ is insensitivity to N fertilizer, i.e., its high yield to a large extent depends on heavy input of N fertilizer. The developed ILs characterized in this study shared much the same GB as HHZ and are phenotypically similar to HHZ but with significantly improved DT under drought stress (Supplementary Tables S1, S2), salt tolerance (Wang et al., 2013b; Pang et al., 2017), submergence tolerance (Wang et al., 2015). However, we have shown that considerable variation remains in the selected ILs for non-target traits such as LNT as indicated in this study, cold tolerance (Zhu et al., 2015) and bacterial blight resistance (Zhang et al., 2015a) in our previous studies. Based on distribution of favorable QTL alleles and phenotypic performance of the target trait such as DT, salt tolerance, submergence tolerance, and non-target trait (LNT) such as cold tolerance, bacterial blight resistance in the selected ILs, DQP was easily carried out to pyramid different favorable alleles of multiple abiotic and biotic stress tolerances by MAS. Many pyramiding lines were selected as expected in this study. Among them, PL6, PL36, PL50, and PL66 with different favorable QTL alleles had better performances in both stresses and normal conditions, which can be directly applied in rice production in drought-prone and/or infertile areas or used as elite parental resources in rice breeding for drought and LN stress tolerances.

## Conclusion

Using three sets of trait-specific ILs in the HHZ background, nine QTL for DT and seven different QTL for LNT-related traits were identified using two different mapping methods. Almost all favorable alleles at the detected QTL were from the three donors. Selection of individuals with different combinations of non-allelic alleles at two DT-QTL (*qDT3.9* and *qDT6.3*), and two LNT-QTL (*qGY1* and *qSF8*) from a pyramiding F_2_ populations derived from HHZ ILs by PCR-based KASP SNP markers, we were able to not only confirm the four detected QTL but also obtain many promising individuals with different DT- and/or LNT-QTL alleles. Four promising lines (PL6, PL36, PL50, and PL66) each pyramiding favorable alleles at two or three QTL were identified, which showed stronger tolerances to drought and/or LN. These lines are being tested in the multi-locational yield trials under drought-prone and/or LN conditions in the rainfed areas, or used for valuable donor parents in rice breeding for improving drought and LN stress tolerances. Our results demonstrated that the trait-specific IL strategy together with high-throughput PCR-based SNP markers is an effective breeding strategy to integrate QTL/allele discovery and breeding application, and that DQP based on phenotypes and genotypes of favorable QTL alleles in ILs is highly efficient and can be widely used in MB for improving complex quantitative traits in the future.

## Author Contributions

JX, WZ, and ZL designed the experiment. BF, KC, YZ, and JA performed all the phenotypic evaluation. YC, BW, and TZ performed analysis and interpretation of the data. BF and WZ drafted the manuscript. JX and ZL revised the manuscript. All authors approved the final version to be published.

## Conflict of Interest Statement

The authors declare that the research was conducted in the absence of any commercial or financial relationships that could be construed as a potential conflict of interest.

## Footnotes

1 http://www.wiserice.com.cn
