# Supplementary material for: Genetic Dissection and Simultaneous Improvement of Drought and Low Nitrogen Tolerances by Designed QTL Pyramiding in Rice
Source: Front Plant Sci. 2018 Mar 9;9:306. doi: 10.3389/fpls.2018.00306 (PMC5855007; doi:10.3389/fpls.2018.00306)
Supplement: Supplementary file 3 [file Data_Sheet_1.DOCX]

**Supplementary Material**

**Segregation distortion method for QTL mapping**

We combined the three selected breeding populations together to perform a joint QTL mapping following the method developed by Cui et al. (2015). As Cui et al (2015) defined, an individual survived from the drought stress with an underlying quantitative trait $y_{j}$called liability which described as the following equation

$$y_{j}=Z_{j}a+\xi_{j}$$

where $Z_{j}$is the genotype indicator for individual *j*, three genotypes are coded as -1, 0 and 1 respectively. $a$is the genetic effect of locus, $\xi_{j}$is the residual error follow the normal distribution $\xi_{j}\sim N(0,1)$. Assume all the individuals survived were screened based on the criterion of$y_{j}>0$. The probability of surviving is the standardized cumulative normal distribution function. The surviving probability of each individual depends on the genotype and the effect of locus $a$. Using the Bayes’ theorem, the posterior probability of survival for each genotype are , , , where and , , are the expected Mendelian frequencies for the three genotypes in BC1F4. Note that when $a=0$, the posterior probabilities are equal to the expected Mendelian frequencies and we will not be able to detect segregation distortion. If $a\neq0$, the posterior probabilities of genotypes will deviate from the expected Mendelian Segregation ratios. Therefore, the segregation distortion loci could be detected by estimating the genetic effect of $a$. For each population, we estimated the effect of each marker and calculated the variance of the estimated effect. Using the Wald test is to estimate the statistics of each marker. For multi-population, the Wald test simply takes the sum of the Wald test of each individual population. The critical value for genome-wide significance at the 0.05 level was drawn from 1000 permuted samples.

**Mixed model approach for QTL mapping**

Association analysis was conducted in the combined three selected populations using a mixed model by treating the founder effects of each locus as fixed effects. The population structure was considered by defining an $n\times4$ matrix of founder allele inheritance indicator for locus $k$ and the kinship matrix was used to estimate the polygenic effect to reduce spurious association (Zhu et al., 2015; Wei and Xu, 2016). Let$y$ be an $n\times1$ vector for the phenotypic values of $n$ individuals and $Z_{k}$ is an $n\times4$ matrix of the founder allelic indicators for locus $k$. The$j\text{th}$row of matrix $Z_{k}$ is a $1\times4$ vector of allelic indicators for individual$j$. If the individual is a homozygote and both alleles from the first founder, the $Z_{jk}$ is defined as $Z_{jk}=[2 0 0 0]$. If the individual is a heterozygote carrying the second and third alleles, the $Z_{jk}$ is defined as $Z_{jk}=[0 1 1 0]$. The sum of all four elements in $Z_{jk}$equals to 2. The mixed model for testing the significance of the$k\text{th}$ marker is defined as

$$y=X\beta+Z_{k}\gamma_{k}+\xi+\varepsilon$$

where $Z_{k}$ is the inheritance indicator for marker $k$, $\gamma_{k}$ is a $4\times1$vector for the four founders allelic effects. $\xi$ is the polygenic effect and $\varepsilon$ is the residual error. The three parameters of this model are estimated by maximizing the restricted maximum likelihood function using Newton iteration algorithm. First, the polygenic effect was estimated under the null model without the marker effect part. Secondly, the effects of the four founders were estimated under the full model. The Wald test was used as the test statistic and P values were obtained from chi-square distribution with 3 degrees freedom. The threshold to declare a significant association was set at a probability level of 1.0×10^-4^ (Matthus et al., 2015; Wissuwa et al., 2015). An LD block harboring significant SNPs was then defined as a putative QTL.
