# Supplementary figures and images for: Genetic Dissection and Simultaneous Improvement of Drought and Low Nitrogen Tolerances by Designed QTL Pyramiding in Rice
Source: Front Plant Sci. 2018 Mar 9;9:306. doi: 10.3389/fpls.2018.00306 (PMC5855007; doi:10.3389/fpls.2018.00306)

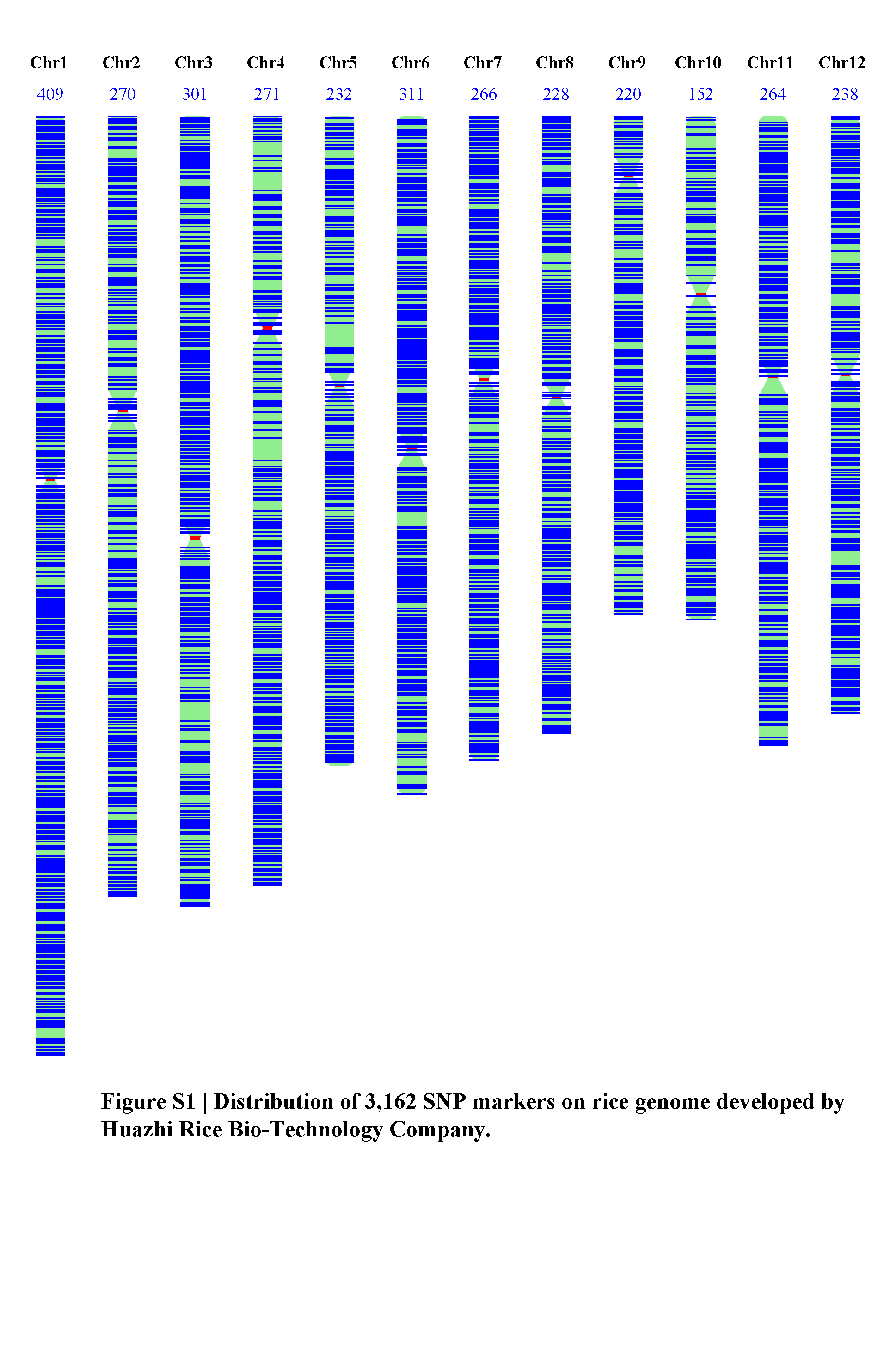

Supplement: Supplementary file 4 [file Image_1.TIFF]
